# Cleavage Specificity Analysis of Six Type II Transmembrane Serine Proteases (TTSPs) Using PICS with Proteome-Derived Peptide Libraries

**DOI:** 10.1371/journal.pone.0105984

**Published:** 2014-09-11

**Authors:** Olivier Barré, Antoine Dufour, Ulrich Eckhard, Reinhild Kappelhoff, François Béliveau, Richard Leduc, Christopher M. Overall

**Affiliations:** 1 Centre for Blood Research, Department of Oral Biological & Medical Sciences, University of British Columbia, Vancouver, BC, Canada; 2 Department of Pharmacology, Faculty of Medicine and Health Sciences, Université de Sherbrooke, Sherbrooke, QC, Canada; 3 Department of Biochemistry and Molecular Biology, University of British Columbia, Vancouver, BC, Canada; NCI-Frederick, United States of America

## Abstract

**Background:**

Type II transmembrane serine proteases (TTSPs) are a family of cell membrane tethered serine proteases with unclear roles as their cleavage site specificities and substrate degradomes have not been fully elucidated. Indeed just 52 cleavage sites are annotated in MEROPS, the database of proteases, their substrates and inhibitors.

**Methodology/Principal Finding:**

To profile the active site specificities of the TTSPs, we applied Proteomic Identification of protease Cleavage Sites (PICS). Human proteome-derived database searchable peptide libraries were assayed with six human TTSPs (matriptase, matriptase-2, matriptase-3, HAT, DESC and hepsin) to simultaneously determine sequence preferences on the N-terminal non-prime (P) and C-terminal prime (P’) sides of the scissile bond. Prime-side cleavage products were isolated following biotinylation and identified by tandem mass spectrometry. The corresponding non-prime side sequences were derived from human proteome databases using bioinformatics. Sequencing of 2,405 individual cleaved peptides allowed for the development of the family consensus protease cleavage site specificity revealing a strong specificity for arginine in the P1 position and surprisingly a lysine in P1′ position. TTSP cleavage between R↓K was confirmed using synthetic peptides. By parsing through known substrates and known structures of TTSP catalytic domains, and by modeling the remainder, structural explanations for this strong specificity were derived.

**Conclusions:**

Degradomics analysis of 2,405 cleavage sites revealed a similar and characteristic TTSP family specificity at the P1 and P1′ positions for arginine and lysine in unfolded peptides. The prime side is important for cleavage specificity, thus making these proteases unusual within the tryptic-enzyme class that generally has overriding non-prime side specificity.

## Introduction

**Figure 1:**
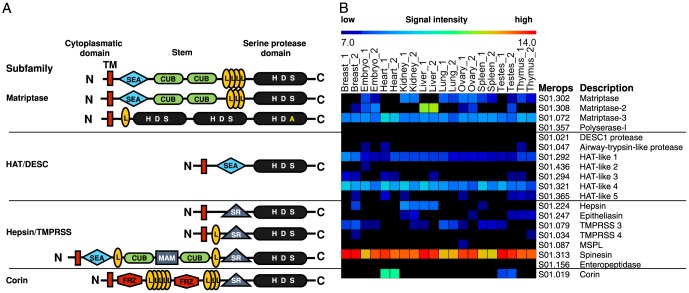
A. Schematic representation of the protein structures and arrangements of the four TTSP subfamilies: HAT/DESC, hepsin/TMPRSS, matriptase and corin. All TTSPs contain a N-terminal transmembrane signal anchor domain (**TM**) and a C-terminal serine protease domain (**H D S**). In case of polyserase-1 one of the 3 catalytic domains is inactive (**H D A**). The stem region of the TTSPs contains 1–6 different domains: the sea urchin sperm protein/enteropeptidase/agrin domain (**SEA**), group A scavenger receptor domain (**SR**), low-density lipoprotein receptor class A domain (**L**), Cls/CLr, urchin embryonic growth factor, bone morphogenetic protein-1 domain (**CUB**) and Corin contains two frizzled (FRIZ) domain. **B.** Murine CLIP-CHIP RNA expression profile and its distribution of 19 members of the TTSP family in 10 murine tissues in duplicates according to their average signal intensity.

Pericellular proteolysis is involved in many important cellular processes such as the transduction of signals across the cell membrane, the release of bioactive growth factors, cytokines and peptide hormones, as well as the interactions with other cells, basement membrane and extracellular matrix proteins [1]–[3]. The increasing relevance of these processes at the cell surface has focused attention on membrane-associated proteolytic systems, including the family of type II transmembrane serine proteases (TTSPs) [4], [5]. The TTSP family is composed of more than 20 members that share a number of structural features including an N-terminal cytoplasmic domain, a transmembrane domain, a central region containing various domains potentially involved in protein-protein interaction, and a C-terminal extracellular serine protease domain (Figure 1A). TTSPs are synthesized as inactive single chain proenzyme zymogens and enzyme activation proceeds by cleavage after a basic amino acid residue in a conserved activation motif N-terminal the catalytic domain. However, the catalytic domain remains membrane-associated after activation because of a disulfide bond that links the prodomain and catalytic domain [6]. Although a few of the TTSPs are expressed across several tissue and cell types, in general these enzymes demonstrate relatively restricted expression patterns, indicating tissue specific regulation or functions [1], [4], [5], [7]–[11].

Based on phylogenetic analysis of the serine protease domains and the domain structure of the extracellular stem region, the TTSPs have been divided into four subfamilies (Figure 1A). The largest is the HAT/DESC subfamily, with currently 7 proteases: human/murine airway trypsin-like (HAT/MAT), differentially expressed in squamous cells carcinoma (DESC)1, and HAT-like 1–5 protease. HAT-like 2 and 3 are only expressed in rodents [12]. HAT is predominantly expressed in the trachea [13], [14] whereas DESC1 is restricted to the epithelia of the skin and oral cavity [15], [16]. TTSPs of the hepsin/TMPRSS/enteropeptidase subfamily include hepsin, mosaic serine protease large form (MSPL), type II transmembrane serine protease (TMPRSS) 2, 3, 4, 5 and enteropeptidase. These TTSPs are expressed predominantly in fetal liver and kidney [1], prostate [17] and on the brush-border of the duodenum, respectively [18]. The matriptase subfamily contains three highly homologous proteases; matriptase, matriptase-2, and matriptase-3, as well as a protein with an atypical mosaic structure, polyserase-1. Matriptase was originally identified from a human breast cancer cell line and shows the most ubiquitous pattern of expression of the TTSP family [9]–[11]. Matriptase-2 plays an important role in iron homeostasis [19]–[21] and is expressed primarily in the liver, where it suppresses hepcidin expression [22]. Matriptase-3 is expressed in brain, ovary, testis and salivary gland [23], whereas polyserase-1 is mainly restricted to skeletal muscle, liver, placenta and heart [24]. The fourth subfamily consists of a single TTSP, corin, which is mainly expressed in cardiomyocytes [25] and is involved in the activation of proatrial natriuretic peptide, a cardiac hormone that regulates blood pressure and cardiac function by promoting natriuresis, dieresis, and vasodilatation [26].

All TTSPs contain a highly conserved catalytic serine protease domain (Figure 1A) of the trypsin (S1) fold subfamily. The large variability of the central modular region together with the diverse expression patterns of TTSP family members suggest that these enzymes may play diverse roles, but few of these functions have been identified. Therefore, substrate discovery is essential to establish *in vivo* functions [27]. As a first step, quenched fluorescence peptide assays combined with positional scanning synthetic combinatorial libraries and phage display approaches have identified a limited number of candidate peptide substrates with their cleavage sites [15], [23], [28]–[31]. However, the protease database MEROPS (http://merops.sanger.ac.uk/) shows that the number of known physiological cleavage sites for the TTSPs is only 52 (+92 synthetic peptide cleavages), and the peptide substrates used only extend from P4 to P4′ [32]. Further, most of these studies do not provide any information of prime side specificity corresponding to the sequence of the carboxyl cleavage product.

By knowing the cleavage site and preferred amino acids in recognition sequences, more specific and selective inhibitors can be developed. To overcome these challenges, we used a proteomics approach to determine the cleavage specificity of six different TTSPs belonging to three subfamilies. PICS (Proteome-wide Identification of Cleavage site Specificity) is a high throughput mass spectrometric approach that allows for the simultaneous identification of the amino acid preference at the cleavage site on both the prime and non-prime sides [33],[34]. This technique using proteome-wide peptide libraries has the possibility to precisely identify several hundred cleaved peptides by tandem mass spectrometry and searchable databases. Since 2008, thousands of new cleavage sites have been both discovered or confirmed using PICS [35]–[39]. In the present study, the amino acid preferences of six human TTSPs, from P6 to P6′, were established from 2,405 cleavage sites and a rational interpretation of family-wide peptide substrate specificity was obtained by linking their specificities to 3D models of their catalytic domains.

## Materials and Methods

### Murine CLIP-CHIP microarray

The expression profile of a panel of 10 commercial murine RNAs (Life Technologies) was analyzed with the murine CLIP-CHIP microarray. mRNA sample preparation for the hybridization to the microarray chip and its analysis were conducted as previously described [40], [41].

### Proteases expression and purification

pRSET5a containing the cDNA of human matripase (UniProt ID Q9Y5Y6) was a generous gift of Dr. U. Bodendorf, Novartis, Switzerland. Human matriptase-2 (Q8IU80) cDNA was a generous gift from Dr. C. Lopez-Otin (Universidad de Oviedo, Oviedo, Spain). Human hepsin (P05981) cDNA was cloned from a human liver cDNA library from Life Technologies. Human DESC1 (Q9UL52) cDNA was a generous gift from Dr. D. E. Schuller (Ohio State University, OH, USA). Human HAT (O60235) cDNA was purchased from GeneCopoeia (Rockville, MD, USA).

Proteases were cloned, expressed and purified as described [29]. In brief, cDNAs corresponding to amino acids 78–811 of matriptase-2, 45–417 of hepsin, 44–423 of DESC1 and 45-418 of HAT were amplified by PCR and ligated into the pMT/BiP/V5-His vector (Life Technologies, Burlington, Canada). These constructs each contained a C-terminal V5-His tag for affinity purification using immobilized metal–chelate affinity chromatography. Drosophila Schneider 2 (S2) cells (Life Technologies) were grown in six-well plates to a density of 2–4×10^6^ cells/mL and transfected with 19 µg cDNAs in pMT/BiP/V5-His and 1 µg pCoBlast selection vector (Life Technologies) using calcium phosphate transfection kits (Life Technologies). The calcium phosphate solution was removed 16 h post-transfection and fresh medium was added. Cells were grown for an additional 2 days. Blasticidin (20 µg/mL) was added to the medium, and the cells were incubated for a 14-day selection period. Cells were then inoculated into 1 L of selection medium and grown to a density of 3×10^6^ cells/mL. Recombinant TTSP expression was induced by adding 500 µM CuSO4. Cells were removed by centrifugation at 6,000×g. Supernatants were loaded on HisTrap FF columns (GE, Mississauga, Canada) and bound TTSPs were eluted using 500 mM imidazole. Fractions were analyzed by immunoblotting and fractions containing TTSPs were pooled and for imidazole removal dialyzed for 16 h at 4 °C against 50 mM Tris-HCl (pH 8.5), 10% glycerol and 250 mM NaCl. Dialyzed pooled fractions were then loaded on HiTrap Benzamidine FF affinity columns (GE, Mississauga, Canada) and eluted with 10 mM HCl (pH 2.0), 0.5 M NaCl and then buffered to 50 mM HEPES (pH 9.0) with 20% glycerol. The four purified TTSPs were active-site titrated with the burst titrant 4-methylumbelliferyl-*p*-guanidino benzoate.

*Spodoptera frugiperda* Sf9 cells expressing mouse matriptase-3 (Q7RTY8) cDNA encoding the serine protease domain (amino acids 581–829) were a kind gift from Dr. T. Bugge (NIH, Bethesda, MD, USA). Matriptase-3 protease was purified as previously described [1], [4], [23] with slight modifications. Conditioned medium was collected from Blasticidin-resistant Sf9 cells, and sodium phosphate buffer (pH 7.5) with NaCl were added to a final concentration of 20 mM and 500 mM respectively. The solution was filtered through a 0.22 µm filter and applied to a HisTrap column using an AKTA explorer system. Chelated proteins were eluted with a linear gradient of 20–400 mM imidazole, and TTSP-positive fractions were identified by immunoblotting using the anti-V5 antibody (Life Technologies). The positive fractions were dialysed overnight at 4 °C against 200 volumes of 50 mM Tris-HCl (pH 8.0), 1 mM CaCl_2_ and 0.1% Tween 20 using dialysis membranes of 10 kDa MWCO.

### PICS analysis

To identify TTSP cleavage sites, we prepared two overlapping proteome-derived peptide libraries. Endoproteinases GluC or chymotrypsin were used to digest HEK293 cell (Life Technologies) proteomes. Peptides were reduced, cysteines alkylated and primary amines protected by dimethylation [33]. The two peptide libraries were then separately incubated with the individual TTSPs after which the N-termini of prime-side cleavage products were tagged with biotin, affinity enriched and identified by liquid chromatography tandem mass spectrometry (LC-MS/MS), performed on a Packings capillary LC system (Dionex) coupled to a quadrupole time-of-flight mass spectrometer (QSTAR XL; Applied Biosystems, Foster City, CA) at the UBC Centre for Blood Research Mass Spectrometry Suite. In brief, samples were resuspended in 5% (v/v) acetonitrile, 3% formic acid and loaded onto a column packed with Magic C18 resin (Michrom Bioresources, Auburn, CA). Peptides were eluted using a 7–40% gradient of organic phase over 95 min. Buffer A was 2% acetonitrile with 0.1% formic acid and buffer B was 98% acetonitrile, 0.1% formic acid. Mass spectrometry data were acquired automatically using Analyst QS software (Applied Biosystems) for data dependent acquisition based on a 1 s mass spectrometry survey scan followed by up to two (QSTAR XL) MS/MS scans of 3 s each. MS and MS/MS data were acquired in centroid mode. QSTAR data were converted to the mzXML format with mzwiff (version 4.3.1). These sequences represent the prime side products of TTSP cleavage. The corresponding non-prime side sequences were derived from human proteome databases using bioinformatics searches. To increase the confidence of our mass spectrometry queries both search engines Mascot (version 2.2.04) [42] and X! Tandem (version 2007.07.01.2) [43] were used and the results were combined. Peptides were analyzed with PeptideProphet [44] and iProphet [45], both implemented within the Trans Proteomic Pipeline (Institute for Systems Biology, Seattle, WA, USA) [46]. Peptides were analyzed at a global false discovery rate of 5% at peptide level by using a decoy search strategy. This allowed for the selection of peptides having a minimum threshold probability corresponding to this false discovery rate percentage [46]. Search parameters included a mass tolerance of 0.4 Da for parental ions and 0.2 Da for fragment ions, allowing up to three missed cleavages, and for the following fixed peptide modifications: carbamidomethylation of cysteine residues (+57.02 Da), dimethylation of lysine amines (+28.03 Da), and variable methionine oxidation (+15.99 Da), variable thioacylation (+88.00 Da) and dimethylation (+28.03 Da) of peptide N-termini. N-terminally thioacylated peptides identified by MS/MS represent prime-side cleavage products of the assayed proteases. The complete cleavage sites were reconstructed by bioinformatic determination of the non-prime side sequences from the human database IPI v3.42 (http://ebi.edu.au/ftp/databases/IPI/last_release/msipi/old/HUMAN/) using WebPICS [39], available at http://clipserve.clip.ubc.ca/pics/. Subsite positions with ambiguous information coming from different protein isoforms were omitted and replaced by X, the one-letter notation for an unknown amino acid. Reconstructed cleavage sites were aligned and summarized as heat maps and iceLogos (University Ghent, Belgium) [7].

### Peptide cleavage site confirmation

In order to confirm the PICS results for the cleavage specificity, mass spectrometry peptide cleavage assays were performed. Scanning through the peptides identified, we selected one peptide sequence that was found for each of the TTSPs analyzed, namely AVIGRKFGDP. The peptide (theoretical mass of 1059.22 Da) was synthesized (GenScript) (>90% purity). For the dimethylated peptide, 30 mM formaldehyde, 30 mM of cyanoborohydride (ALD coupling solution, Sterogene, Carlsbad, CA) and 10 mM HEPES pH 7.4 were added to the peptide and incubated overnight (18 h) at room temperature to generate (dm)AVIGR(dm)KFGDP. To assay for cleavage, 200 µg of AVIGRKFGDP or (dm)AVIGR(dm)KFGDP were added to 2 µg of matriptase, matriptase-2, HAT and hepsin in 100 mM HEPES, pH 7.4, 150 mM NaCl and incubated at 37°C for 18 h. 20 µL of the reaction mixtures were used and buffer exchanged using C18 OMIX 10 µL tips (Agilent, Mississauga, Canada) using the manufacturer's instructions. Samples (0.8 µL) were mixed with 0.5 µL of 10 mg/mL of CHCA matrix solution (Sigma, Oakville, Canada) and spotted on a stainless steel MALDI plate. Spectra were acquired in reflectron positive mode and 2,175 random shots were performed and analyzed on a 4700 MALDI-TOF/TOF mass spectrometer (Applied Biosystems) with a mass range of 300-1,200 Da. Data analysis was conducted using protein data software provided by Applied Biosystems.

### Structural analysis

For matriptase, hepsin, and DESC1, the crystal structures 3p8g [47], 1z8g [48] and 2oq5 [49] were used, respectively. For matriptase-2, -3 and HAT, structural models were generated using the interactive mode of the modeling server SWISS-MODEL [50], [51], and crystal structures of closely related enzymes identified by the FFAS03 server [52], [53]. We used DESC1 (PDB code 2oq5) [49] for matriptase-2 and HAT, and matriptase (PDB code 3p8g) [47] for matriptase-3. Sequence numbering was corrected and secondary structures assigned to all structures and models using the UCSF Chimera package [44]. The topology representation was created using TopDraw [54]. Peptide docking with the structural model of matriptase-2 was performed using the Rosetta FlexPepDock web server [55], [56], allowing full flexibility to the peptide (AEAALR(P1).(P1′)KLLEVA), and full side-chain flexibility to the protease. Initial models were based on available complex structures of matriptase with aprotinin (PDB code 1eaw) [47] and trypsin inhibitor 1 (PDB entry 3p8f) [57], and our PICS P6 to P6′ sequence results. Molecular graphic figures were made using the molecular visualization system PyMOL (The PyMOL Molecular Graphics System, Version 1.5.03, Schrödinger, LLC) [58].

## Results

### Tissue distribution of TTSPs

The tissue distribution of TTSP family members was determined by transcript analysis of ten murine tissues using the dedicated murine CLIP-CHIP [1]–[3], with probes for all murine proteases, inactive homologues, and inhibitors (Figure 1B). The expression profile reveals that most of the TTSPs are very low or non expressed (average signal intensity below 7.5) in the analyzed tissues with the exception of the high expression of matriptase-2 in liver and corin in heart, both of which have been previously described as predominantly expressed in these tissues [25], [28]. Hepsin shows significant expression in liver and kidney, TMPRSS3 in breast and lung, matriptase in kidney and lung. Matriptase-3, Hat-like 1, Hat-like 4 and spinesin show expression in all 10 tissues. However, polyserase-1, DESC1 and enteropeptidase were not detected in these ten tested tissues. This suggests potential tissue specificity for some of the TTSPs whereas other TTSPs might be involved in more general processes due to their ubiquitous expression.

### Sequence alignment and structural analysis of the catalytic domain of TTSPs

**Figure 2:**
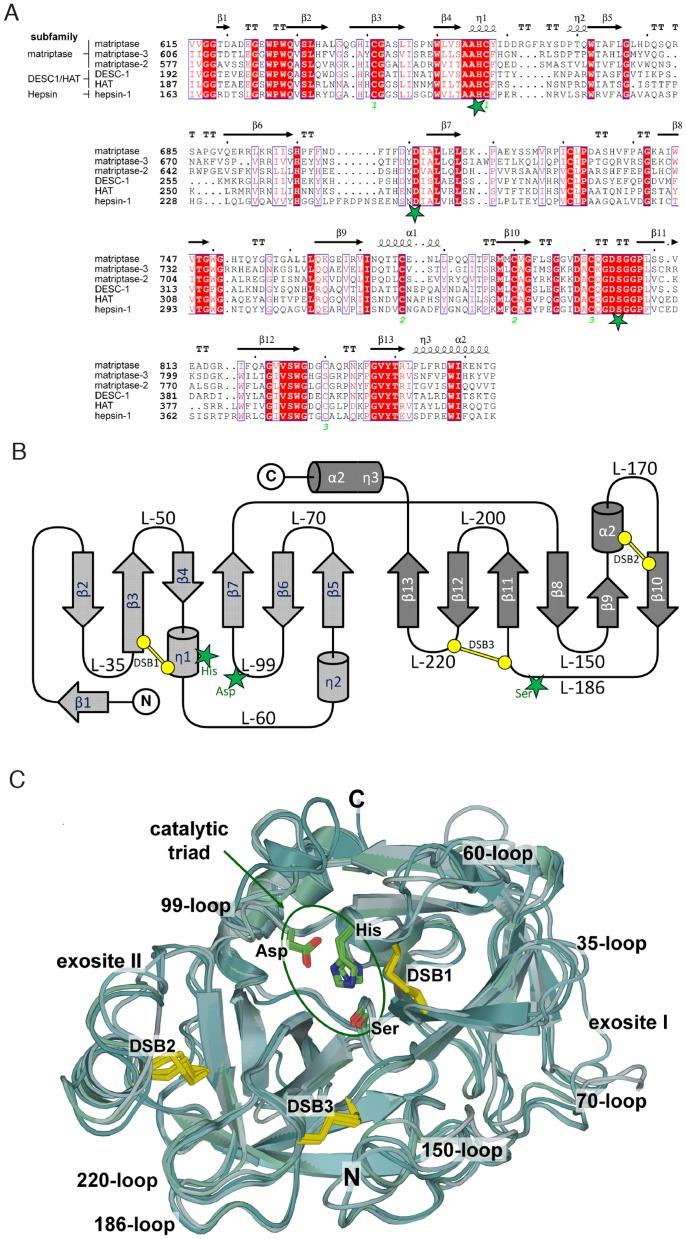
A. Multiple sequence alignment of the catalytic domains of six TTSPs was performed with Clustal Omega [59] and displayed using ESPript 2.2 (http://esprit.ibcp.fr/ESPript/cgi-bin/ESPript.cgi [63]. Green stars indicate the residues of the catalytic triad; conserved residues are shaded in red. Blue boxes indicate amino acid with similar physico-chemical properties. Secondary structure elements are represented with flat arrows for β sheets and helices for α- and 3_10_- (η) helices. Three conserved disulfide bridges are indicated in orange numerals below the alignment. Sequence numbering is according to the respective UniProt entries. **B.** Topology diagram of a TTSP catalytic domain. Residues of the catalytic triad are indicated as green stars, disulfide bonds (DSB) are shown in yellow. Loops (L-) are labeled corresponding to the thrombin nomenclature suggested by Bode et al. [64]. **C.** Superposition of the catalytic domains of matriptase, matriptase-2, matriptase-3, hepsin, DESC1 and HAT, including both known and modeled structures, in traditional serine protease standard orientation [65]. Catalytic triad and conserved disulfide bridges are shown in ball and stick representation. Important loops and exosites are indicated and labeled.

To better understand the cleavage specificity of the TTSP family, we first compared the sequences of their catalytic domains using Clustal Omega [59] and ESPript 3.0 [57] (Figure 2A). The catalytic domain of matriptase showed the highest sequence identity with matriptase-2 (45% identity) and matriptase-3 (31% identity). HAT showed the highest sequence identity with DESC1 (50% identity). From the three subfamilies, the conserved regions within the catalytic domain are clearly visible (indicated in red). The catalytic triad, indicated as green stars, consists of histidine 656, aspartate 711 and serine 805, following the numbering of matriptase (top row). Interestingly, strictly conserved regions are often observed in stretches that adopt a β-sheet structure (Figure 2A). A topology diagram of a typical TTSP catalytic domain is shown in Figure 2B. Conservative substitutions, for example such as leucine by valine and serine by threonine were identified in the β4-strand close to the active site. This suggests that a specific chemical environment is necessary for the catalytic domain to perform catalysis.

Such conservation of key regions and three disulfide bridges led us to hypothesize that the catalytic domain of all the TTSPs had structural similarities. Crystal structures of matriptase [47], DESC1 [49] and hepsin [48], [60] have been solved previously. Therefore, matriptase-2, matriptase-3 and HAT were modeled and analyzed by superimposition of all the catalytic domains (Figure 2C), and typical structural elements of serine proteases of the trypsin fold are depicted. The first striking aspect observed was that the structures of all the TTSP catalytic domains are similar and highly superimposable with few major differences. One of these differences occurs in matriptase-3 where an extended loop in its catalytic domain following the β7 strand that is diverging from the other family members. This loop may interact with substrates or effectors that could induce a conformational change in the matriptase-3 catalytic domain and modulate substrate binding and presentation to the active site for catalysis. Another example is the extended 60-loop between the two 3_10_-helices (η1 and η2) of matriptase, which potentially provides additional substrate specificity and possibly flip over the active site upon substrate binding, blocking further access until cleavage occurs. However, these differences in structure do not affect the active site geometry of the catalytic triad in these TTSPs, and their specificity for peptidic substrates, but may explain differences in native substrate cleavage or in comparing peptide cleavage specificities with native protein cleavage specificities.

### PICS analysis represented by heat maps and iceLogo

Using PICS, 2,405 cleavage sites were identified using both chymotryptic and GluC-generated proteome-derived peptide libraries, greatly expanding upon the known 144 cleavage sites annotated in MEROPS (Tables S1–S12 in File S1). These peptide libraries were selected because the TTSP family belongs to the trypsin-like family and so have a lysine or arginine specificity in P1. Thus, peptide libraries generated by trypsin for use with the PICS technique will contain peptides displaying an arginine or lysine at the C-terminus and hence will not contain internal basic residues. In such cases alternate peptide digestive proteases are easily used to generate appropriate peptide libraries. Here we used chymotryptic and GluC peptide libraries in which the C-terminal residues were aromatic/hydrophobic or negatively charged, respectively. These two libraries are overlapping and both libraries display internal lysine and arginine residues and complement each other by displaying acidic and hydrophobic residues within the peptide sequences. Using two or more libraries also allows for experimental validation of results obtained from one library with the other.

**Figure 3:**
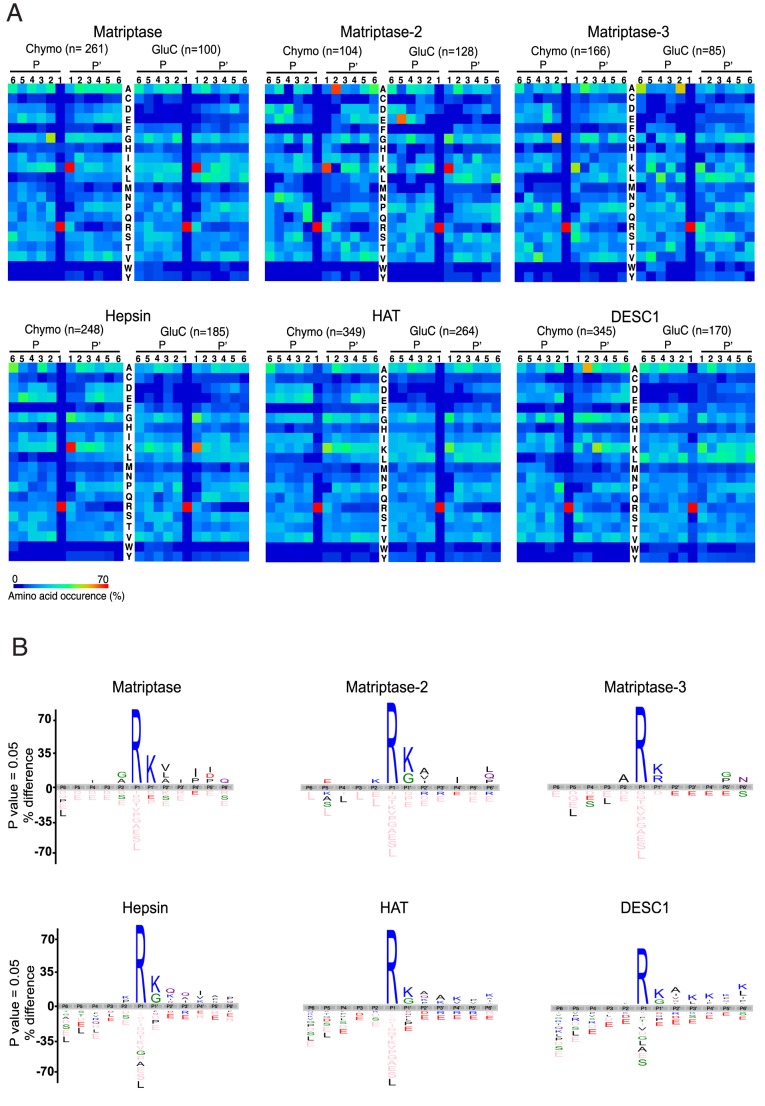
A. Matriptase, matriptase-2, matriptase-3, hepsin, DESC1, an HAT cleavage site specificities obtained by PICS using two different peptide libraries: GluC and chymotryptic. For each class of peptide library, the amino acid occurrences in P6–P6′ are shown as heat maps. Above each heat map, the number of identified cleaved peptides is specified. **B.** Taking into account the relative natural abundances of amino acids, iceLogo representations of the different cleavage site specificities obtained from the GluC library are shown. Peptide sequences were aligned on the scissile bond between P1 and P1′. Statistically significant amino acid residue occurrences present (P<0.01) were plotted and amino acid heights are indicative for the degree of conservation at the indicated position. Residues that were completely absent in the identified terminal peptides are shown below in pink.

From the heat maps generated for the six human TTSPs, a common feature was apparent—arginine is preferred in the P1 position, ∼17-fold more than the natural occurrence of arginine in proteins, and lysine is preferred in P1′ (Figure 3A). Note in the PICS workflow lysine is dimethylated. For several TTSPs, the P1′ position has glycine as an alternative amino acid preference, which is strongly favored in the case of hepsin. Similar specificities also occur within subfamilies. For example, for matriptase and matriptase-3, glycine or alanine is important at P2, whereas matriptase-2 prefers glutamate in P5 and alanine in P2′. Another example is across the HAT/DESC1 subfamily, where alanine and lysine are preferred in the P2′ and P3′ positions, respectively, for DESC1, but these are less preferred for HAT. HAT requires an aspartate in P2, a feature that was shown by Wysocka et al [31] using positional scanning libraries.

The iceLogo representation (Figure 3B) also revealed the least preferred amino acid preferences at each position. For example, since arginine is the only preferred amino acid at P1, this means that all other residues are thereby under represented. Similar negative preferences result from the lysine or glycine in P1′. Least preferred residues also reinforces the differences among members of the same subfamily. For example, matriptase and matriptase-3 prefer a glycine in P2, but matriptase-2 does not. Conversely, matriptase-2 favors a glutamate in P5, but this is not the case for matriptase and matriptase-3. This also points out the utility of PICS as it is easy to profile 6 amino acid residues in both the non-prime and prime sides, which other techniques generally do not do cover. Another interesting finding was the preference for alanine in the P2′ position for most of the TTSPs, especially important for matriptase-2 and DESC1, suggesting a specific feature for these two subfamilies.

**Figure 4:**
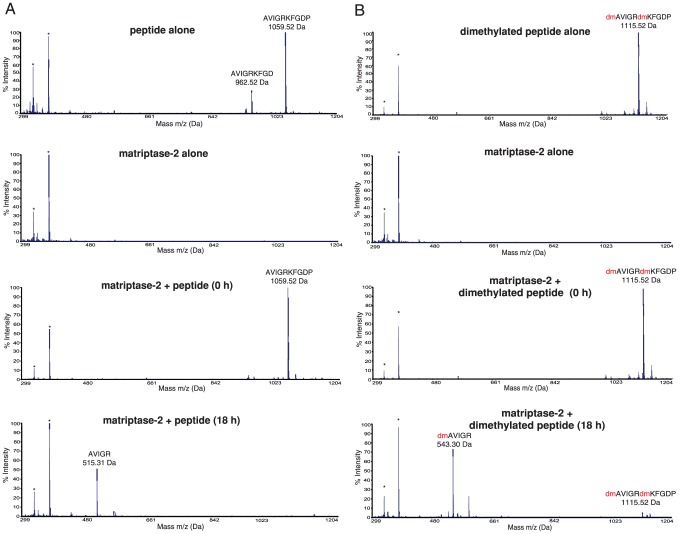
**A. *Upper panel*.** MALDI-TOF spectrum of the synthetic peptide AVIGRKFGDP. The sample contained a minor synthesis contaminant of AVIGRKFGD. The experimental determined [M+H]^+^ is 1059.52 Da (predicted m/z is 1059.22 Da). The sequence is presented by the spectra. The two asterix peaks represent the MALDI matrix peaks which are found in all the spectra in this m/z range. ***Second panel.*** MALDI-TOF spectrum of matriptase-2. ***Third panel.*** MALDI-TOF spectrum of the synthetic peptide added to matriptase-2 at 0 h. ***Lower panel.*** MALDI-TOF spectrum of the assay reaction products generated after incubation of the synthetic peptide with matriptase-2 for 18 h. The spectral peak at 1059.52 m/z disappeared and a new peak at 515.31 Da appeared corresponding to the cleavage product [AVIGR+H]^+^ (predicted m/z is 514.96 Da). **B.** ***Upper panel.*** MALDI-TOF spectrum of the dimethylated (dm) synthetic peptide (dm)AVIGR(dm)KFGDP. The experimental determined [M+H]^+^ is 1115.52 Da. The sequence is presented on the spectral peak. The two asterix peaks represent the MALDI matrix peaks and they can be found in all the spectra. ***Second panel.*** MALDI-TOF spectrum of matriptase-2. ***Third panel.*** MALDI-TOF spectrum of the reaction products after incubation of the dimethylated synthetic peptide with matriptase-2 added at 0 h. ***Lower panel.*** MALDI-TOF spectrum of the reaction products after incubation of the dimethylated synthetic peptide with matriptase-2 for 18 h. The peak at 1115.52 Da disappeared and a new peak at 543.30 Da appeared, corresponding to [dAVIGR+H]^+^. Red (dm) is dimethylation.

To confirm the identified cleavage specificities of the six studied TTSPs we used synthetic peptides. We first examined the PICS mass spectrometry results in more detail and found that the AVIGRKFGDP peptide ([M+H]^+^  =  1059.52) was cleaved by all TTSPs. This peptide was synthesized and incubated with matriptase-2 (Figure 4), matriptase, HAT and hepsin (Figures S1–S3), and the cleavage site was identified by MALDI-TOF MS. The N-terminal nonprime cleavage product AVIGR (515.30 Da) was identified after cleavage between Arg and Lys confirming the unusual P1′ specificity.

In PICS, all primary amines within the peptide library are dimethylated resulting in a dimethylated lysine side chain with altered charge, raising the question about the involvement of lysine in P1′. We found that the dimethylated (dm) version of the peptide (dm)AVIGR(dm)KFGDP was also cleaved by matriptase-2 (Figure 4B) and all other tested TTSPs with similar kinetics, validating the identified PICS cleavage sites for these TTSPs. Overall, our data reveal that the prime side is also important for the cleavage specificity of the TTSPs where assayed using unstructured peptide libraries. This makes the TTSPs unique within the tryptic-enzyme class that are dominated by P1 interactions.

### Interpreting cleavage specificity by structural modeling of matriptase-2

**Figure 5:**
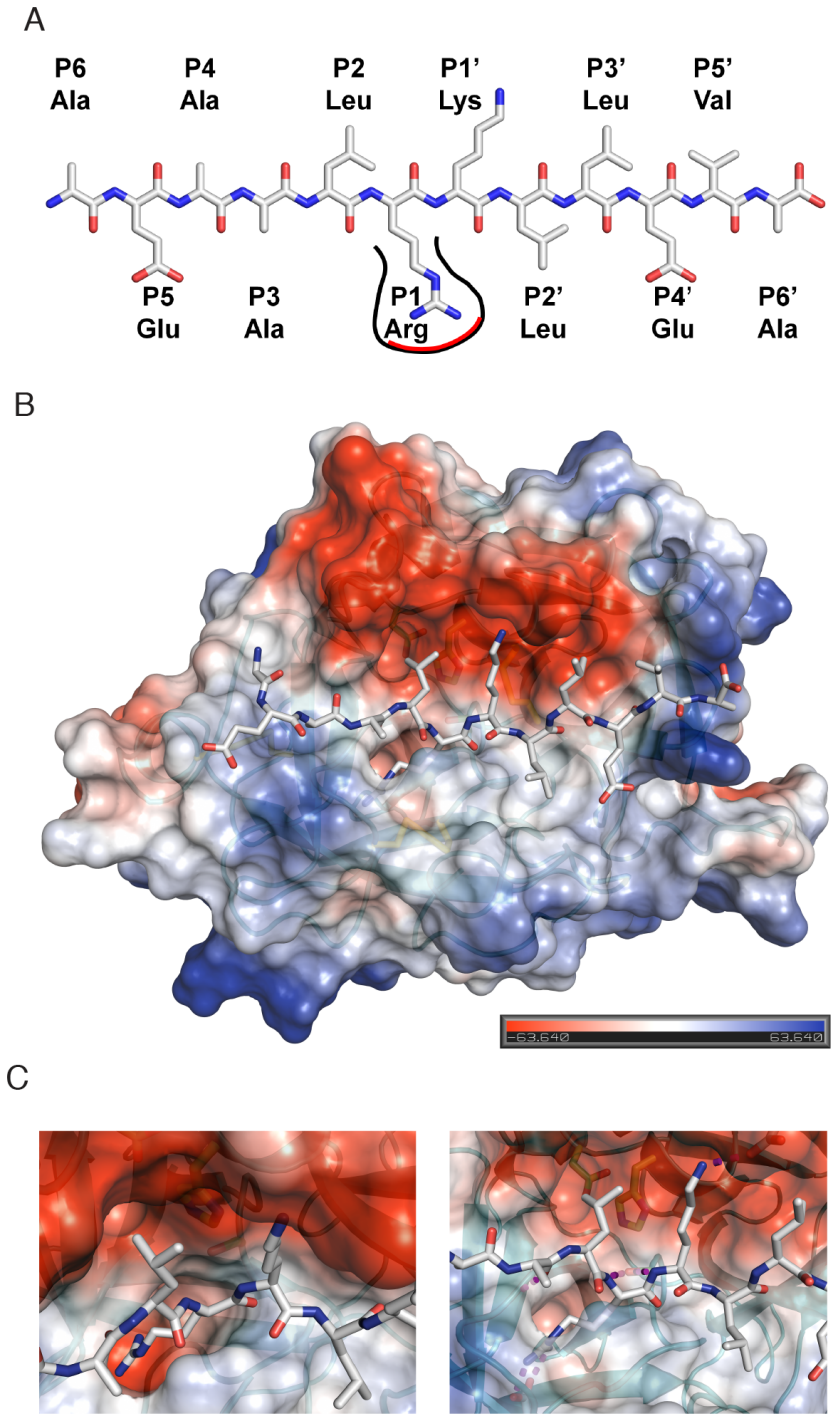
A Schematic representation of the dodecapeptide (AEAALR↓KLLEVA) used for active site docking. The deep TTSP S1 subsite, accommodating the P1-Arg, is shown. **B**. Structural model of matriptase-2 with the peptide substrate (AEAALRKLLEVA) identified by PICS docked into the active site. Matriptase-2 was colored according to its surface charge distribution, and is shown in standard orientation with the modeled peptide occupying subsites S6 to S6′ (from left to right). **C**. Close-up views of the active site illustrating the deep and negatively charged S1 pocket (left) and key stabilizing interaction between the protease and the modeled peptide (right).

Based on the known crystal structures, a matriptase-2 3D homology model was generated (Figure 2C and Figure 5), and a PICS determined model peptide, AEAALR↓KLLEVA (Figure 5A) was incorporated in the active site by peptide docking. An electrostatic surface charge representation of matriptase-2 including the docked peptide is shown in Figure 5B, illustrating the negative charge distribution of the upper half of the active site including subsite S2 and S1′, and the slightly positive subsites S5 and S4′, explaining their opposed preferences for charged residues. The deep S1 pocket with Asp756 forming the negative base and main interactor of the P1-Arg is depicted in Figure 5C. Both the groove and the electrostatic environment suggest that a positively charged amino acid composed of a long side chain can be readily accommodated and stabilized in S1. Indeed, this would explain why an arginine is highly favored at the P1 position. Noteworthy, both backbone nitrogens of the P1 arginine and the P1′ lysine are hold in position by the active site Ser762. Specificity for lysine in P1′ is mainly provided by the negatively charged side-chain of Glu621 at the top of the S1 subsite (Figure 5C). We suggest that these features are the key players for the preferred cleavage between arginine in P1 and lysine in P1′.

Glycine 784 of the protease backbone connects to the P2 position of the peptide through hydrogen bonding and stabilizes this atom at the non-prime site of the peptide. The slight preference in this position for glycine might be guided by the high flexibility of its α carbon, which may compensate for less-preferred amino acids in other subsites. Subsite P3′, which favors small hydrophobic residues for most of the investigated TTSPs, is stabilized by anti-parallel backbone-backbone interaction of the peptide to the protease.

## Discussion

By utilizing diverse natural peptides of variable length and amino acid composition, PICS identifies the cleavage specificity of a protease and the exact sequence on both sides of the scissile bond by analysis of hundreds of cleaved peptides in each experiment [33], [34]. The mass spectrometric determination of the active site specificity of six important members of the TTSP subfamilies using PICS revealed several important features. First, it is an unusual finding for tryptic proteases for their specificity to be determined by both the P1 and P1′ positions. Second it is also unusual for tryptic proteases to have specificity exerted through extended features on the prime side. The specificity for arginine in P1 is in agreement with other recent reports using other techniques such as positional scanning libraries and/or fluorescent substrates [15], [23], [29], [31], [60], [61]. Furthermore, almost all of the known protein substrates of the assayed TTSPs contain an arginine in P1 position (Table 1). However, we cannot rule out that native substrates with a lysine in P1 might be cleavable since all lysines in PICS are dimethylated to protect against NHS-biotin reactivity, which is used to selectively tag the neo-N terminal α-amine for enrichment after cleavage. Third, the specificity for lysine in P1′ is shown for the first time using a peptide library technique in our study and is consistent with the natural substrate specificity of matriptase, which cleaves for example CDCP1 and laminin, alpha 1-like between arginine and lysine (Table 1). Consistent with our findings, Beliveau *et al* [29] demonstrated that negatively charged amino acids are the least preferred at P1′ position as shown by having a very low k_cat_/K_M_.

**Table 1:** Known protein substrates obtained from MEROPS [32] and UniProt [66] showing the P4-P4′ cleavage specificity in natural substrates of matriptase, matriptase-2, matriptase-3, HAT, hepsin and corin where cleaved as native proteins *versus* denatured peptides in PICS.

				Cleavage site					
Matriptase substrates	P4	P3	P2	P1	P1′	P2′	P3′	P4′	References
CDCP1	Lys	Gln	Ser	Arg	Lys	Phe	Val	Pro	[67]
fibronectin	Val	Thr	Gly	Arg	Gly	Asp	Ser	Pro	[68]
filaggrin	Arg	Lys	Arg	Arg	Gly	Ser	Arg	Val	[11]
filaggrin	Thr	Gly	His	Gly	Gln	Ala	Ser	Ser	[69]
hepatocyte growth factor	Lys	Gln	Leu	Arg	Val	Val	Asn	Gly	[11]
insulin-like growth factor-binding protein 1	Lys	Ala	Leu	His	Val	Thr	Asn	Ile	[11]
insulin-like growth factor-binding protein 7	Arg	Lys	Gly	Lys	Ala	Gly	Ala	Ala	[70]
laminin, alpha 1-like	Arg	Val	Val	Arg	Lys	Gly	Val	Ser	[68]
matriptase	Arg	Gln	Ala	Arg	Val	Val	Gly	Gly	[11]
prostasin	Ile	Gln	Pro	Arg	Ile	Thr	Gly	Gly	[71]
proteinase-activated receptor 2	Ser	Lys	Gly	Arg	Ser	Leu	Ile	Gly	[11]
stromelysin-1	Arg	Lys	Pro	Arg	Cys	Gly	Val	Pro	[72]
urokinase-type plasminogen activator	Pro	Arg	Phe	Lys	Ile	Ile	Gly	Gly	[11]
**Matriptase-2 substrates**									
matriptase-2 precursor	Pro	Ser	Ser	Arg	Ile	Val	Gly	Gly	[73]
matriptase-2	Pro	Gly	Val	Arg	Val	His	Tyr	Gly	[73]
hemojuvelin	Ile	Ile	Ile	Arg	Gln	Thr	Ala	Gly	[74]
**Matriptase-3 substrates**									
alpha-2-antiplasmin	Ala	Met	Ser	Arg	Met	Ser	Leu	Ser	[23]
antithrombin-III	Ile	Ala	Gly	Arg	Ser	Leu	Asn	Pro	[23]
heparin cofactor 2	Phe	Met	Pro	Leu	Ser	Thr	Gln	Val	[23]
plasma serine protease inhibitor	Phe	Thr	Phe	Arg	Ser	Ala	Arg	Leu	[23]
plasminogen activator inhibitor-1	Val	Ser	Ala	Arg	Met	Ala	Pro	Glu	[23]
**Hepsin substrates**									
coagulation factor IX	Asp	Phe	Thr	Arg	Val	Val	Gly	Gly	[60]
coagulation factor VII	Pro	Gln	Gly	Arg	Ile	Val	Gly	Gly	[75]
coagulation factor XII	Ser	Met	Thr	Arg	Val	Val	Gly	Gly	[60]
hepatocyte growth factor	Lys	Gln	Leu	Arg	Val	Val	Asn	Gly	[60]
hepsin precursor	Pro	Val	Asp	Arg	Ile	Val	Gly	Gly	[60]
laminin subunit beta-3	Ser	Gln	Leu	Arg	Leu	Gln	Gly	Ser	[61]
urokinase-type plasminogen activator	Pro	Arg	Phe	Lys	Ile	Ile	Gly	Gly	[76]
**Corin substrates**									
atrial natriuretic peptide-converting enzyme	Ser	Val	Val	Arg	Asn	Met	Glu	Met	[77]
atrial natriuretic peptide-converting enzyme	Gly	Asp	Gln	Arg	Cys	Leu	Tyr	Asn	[77]
pro-atrial natriuretic peptide	Thr	Ala	Pro	Arg	Ser	Leu	Arg	Arg	[18]
**HAT substrates**									
urokinase plasminogen activator surface receptor	Asn	Ser	Gly	Arg	Ala	Val	Thr	Tyr	[78]
urokinase plasminogen activator surface receptor	Thr	Tyr	Ser	Arg	Ser	Arg	Tyr	Leu	[78]

PICS showed that if the nature of the peptide substrate was negatively charged, non-polar, or hydrophobic, then these characteristics affected the amino acid preferences accommodated in the TTSP active sites depending on which library was used. For example, using a chymotryptic library, a preference for aspartate and glutamate was identified at the P3, P4 and P5 positions for hepsin, matriptase, matriptase-3 and DESC1, whereas peptides generated by GluC had a leucine and/or lysine preferred at P3 and P4. Since aspartate and glutamate are displayed internally in the chymotryptic peptides these libraries better reflect the true specificities for acidic residues than can be derived from GluC libraries, which are internally depleted for negatively charged residues. This reaffirms the need for using at least two libraries in PICS to accurately interpret cleavage specificity preferences for proteases [33], [34].

It was notable that the prime side of a peptide is important for TTSP cleavage specificity. Thus alanine or other small hydrophobic residues are required in the P2′ position, as well as nonpolar amino acids such as leucine and valine at the other prime positions. This feature can also be observed in natural substrates of matriptase (hepatocyte growth factor), matriptase-3 (plasminogen activator-inhibitor-1) and hepsin (coagulation factor IX and VII) (Table 1).

The application of PICS was shown to be highly successful by the identification of 2,405 cleavage sites for the six TTSP family members studied. These data greatly expand the 144 known cleavages sites for these proteases. The specific amino acid residue features found in our study of three subfamilies of the TTSPs provide new insight that might be used to design specific inhibitors and peptide substrates, such as AEAALR↓KLLEVA that we identified here and validated. It is important to keep in mind that the overall similarities in specificity for the TTSPs will likely render small molecule drug development challenging. The presence of several domains outside the catalytic domain suggests that these exosites could be critical for substrate binding and recognition [62] and undoubtedly influence native substrate binding and cleavage of intact proteins. Indeed many native protein substrates do not have Lys in P1′. Native protein structures often mask even highly preferred cleavage sites rendering these unavailable for protease access and cleavage. However, this can be levered to advantage in designing small molecule inhibitors. Thus future studies of exosite interactions, and possibly designing exosite inhibitors, will be insightful in revealing TTSP specificity for natural substrates *in vivo* and hence *in vivo* roles.

## Supporting Information

Figure S1 (EPS)

Figure S2 (EPS)

Figure S3 (EPS)

File S1 Tables S1-S12.
(XLS)
